# On a Case of Cerebral Syphilis; with Consideration of the Diagnosis of Lesion of the Corpora Quadrigemina

**Published:** 1876-11

**Authors:** 


### Translations.

### ON A CASE OF CEREBRAL SYPHILIS; WITH CONSIDERATION OF THE DIAGNOSIS OF LESION OF THE CORPORA QUADRIGEMINA.

*Translated from the Italian of Fiori,**

BY JAMES NEVINS HYDE, M.D.

On the seventeenth day of last February, Ricolfi Alessandro presented himself at the Medical Clinic of the Royal University of Turin. He was 33 years old, unmarried, and had been previously engaged in the provision business. At the time of his first appearance in the Clinic, while he was standing upon his feet, and the professor, in the presence of his assistants and students, was engaged in eliciting the facts concerning the previous history and actual condition of the patient, the latter

* Intorno a un caso di sifilide cerebrale per riguardo alla diagnosi della lesioni delle eminenze quadrigemelle; del Dott. Gian Maria Fiori, 3d Assistente alla clinica del Prof. Rovida a Torino. (*Giornale della R. Accademia di Medicina di Torino. Aug.* 10, 1876.)

was attacked by a very peculiar convulsive seizure. His neck was forcibly twisted to the left, to the extent of about one-fourth of its circumference; and at the same moment his entire body was rotated several times from the right to the left, as it were revolving upon its longitudinal axis. This attack lasted for but a few minutes, when the patient became completely restored to his former condition, and, having assured himself that all was over, continued to respond to the interrogatories that were put to him.

He was a man of good constitution, with a properly shaped cranium, and exhibited no anomaly of the osseous framework or the muscular system, which supported it. His intellectual faculties were unimpaired, and his words were accompanied by energetic and expressive gestures. He stated that his parents had enjoyed robust health, and that he had not suffered from hereditary disease. He had not been affected by any malady in infancy or childhood — nothing, in fact, had occurred to disturb the heedless life of pleasure which he had for some time led, though he had paid his devotions both to Bacchus and Venus. In 1863, however, he contracted a blennorrhagia, which in its course became complicated with orchitis. It was treated by baths containing various mineral and vegetable ingredients, and was only cured after relapses. No treatment was adopted for the relief of the discharge, which was, at the outset, quite severe, the result being that spontaneous cure resulted in from four to five months, leaving a urethral stricture of moderate degree. He suffered from sore throat during the ensuing year, which his attending physician considered to be a manifestation of syphilis, and treated with pencillings of the nitrate of silver, and the administration, internally, of pills (probably mercurial) of the nature of which the patient was ignorant. In 1866 he had manifestations in the vicinity of the anus, which the physician pronounced to be syphilitic mucous tubercles. These lesions disappeared in from fifteen to twenty days, after cauterization with the nitrate of silver, without internal medication. During the Carnival of 1873, he received a stroke with a sabre, in the posterior part of the left parietal region, near the sagittal suture. The resulting wound was healed in ten days without grave consequences, leaving a cicatrix in the region named, which is still visible. It is linear, slightly depressed, two and a half centimetres long, and is directed obliquely from above downward, and from behind forward. In May, 1874, without any known cause and with no prodromata, he suddenly fell to the earth while taking a walk in the country. He was then unconscious, and had clonic contractions of the entire body, particularly of the left side, with marked traction of the left labial commissure toward the ear. This attack lasted for about ten minutes, resulting, however, in paralysis of the left arm and leg, which disappeared in eight days after blood-letting and blistering. One month afterward he suffered from another similar attack, and others followed at a like interval, until very recently, when the accesses became so frequent that they disqualified him from attending to any business. After the first two attacks, however, there was no loss of consciousness, and, up to the date of his admission to hospital, the contractions were generally limited to the muscles of the left side of the neck and face.

On the evening after his admission (February 17) the spasmodic attacks were repeated with unusual severity and frequency. Each was preceded by a peculiar feeling of faintness, which warned the patient of the approaching crisis, and gave him time to take precautions for his safety. It began with tonic contraction of the right sterno-cleido-mastoid muscle, thus the head was turned to the left; then suddenly followed tetanic contractions of the eyelids of both sides and the muscles of the cheek of the left side, with tonic contraction of the muscles of the left side of the neck, including the sterno-cleido-mastoid of that side. Almost at the same time could be noticed contractions, at first clonic and then tonic, of the left limbs, with slight and transient contractions of the right arm and leg. During the access the head remained forcibly twisted to the left, and somewhat inclined upon the side, notwithstanding the fact that the right sterno-cleido-mastoid, contracted at the outset, yielded and became flaccid as soon as the attack was precipitated. The globes of the eyes at this time were entirely concealed by the contracted lids; upon the separation of the latter, both eyeballs could be seen turned upward and to the left, their pupils being dilated. The left labial commissure was forcibly dragged toward the ear of the same side. The condition of the tongue, velum and uvula could not be observed. The pulse and respiration remained normal, and the condition of general sensibility almost normal. Although unable to speak, he was conscious of his condition, and heard what was said to him.

Each convulsion lasted from two to five minutes, and if, during its continuance, an attempt was made to turn the head to the right by decreasing the muscular contraction, the access was somewhat shortened. On the occasion of the first attack which happened after his admission to hospital, the patient had been seized with motor paralysis of the left arm and leg, with consequent flaccidity of the muscles supplying these limbs, and paresis of those of the left side of the face. There was also slight ptosis of the left upper eyelid. The frontal rugæ were pronounced on the right side, and absent on the left — on this latter side, also, there was a feeling of tumefaction of the cheek, and partial failure of movement of the jaw in the act of mastication.

Finally, after each attack, the patient could describe in words the sensations he had just experienced, and could move his head as he pleased. His eyes returned to their normal position, the pupils responding properly to the light. The traction upon the left labial angle disappeared; his tongue could be projected in the median line, and there was no deviation of the uvula or velum. General sensibility became also completely restored, with the exception of a sensation of pricking and formication in the left arm and leg.

The lesions of the cerebral nerves above described evidently pointed to some disease of the brain, and the complexity of phenomena demonstrated that its location was to be sought within certain limits.

Difficult as it may at first sight appear to determine the site of lesion, some of the symptoms point to it with sufficient distinctness. These are: the movement of rotation upon one axis, the deviation of the eyes to the same side, and the perfect hemiplegia of the limbs and face of that side, consecutive to tonic and clonic contractions.

But while the last corresponds to the ordinary form of hemiplegia from cerebral hæmorrhage involving the corpora striata, the optic thalami and the lenticular nucleus, it excludes the possibility of a lesion of the base of the brain, which would be accompanied by alternate paralysis of the face and limbs. The movement of rotation and the deviation of the eyes, on the other hand, when considered together, may be referred to lesion of a cerebellar peduncle, a cerebral peduncle or the corpora quadrigemina.

In order to arrive at a solution of the question which, stated in this manner, may seem exceedingly intricate, it will be well to notice, first, that we are not in position to form an exact idea of the nature and mode of the rotary motion observed in our patient. This movement, in point of fact, could be studied only upon one occasion, that viz., when the patient first entered the clinic, because, on that same day, the paralysis began to be declared which soon rendered the vertical position — and of course walking — impossible. During this single attack, however, the patient was observed in the act of walking, though the deviation of the eyes could not be noted; but the period of access was so brief that we had not time to determine what muscles were most interested in the phenomena. It was only possible to establish the deviation of the head to the left, and the twisting of the patient upon his left flank, with a restricted turning (almost equal to a rotation) upon his vertical axis.

These two facts, however, readily recall to mind the rotary movements obtained by Longet, Magendie, Schiff and others, in animals with lesion of the middle cerebellar peduncles; while they render it as less probable, that we had witnessed the movement of the circus-rider, observed by Magendie after lesion of the cerebral peduncle and the optic thalami. Such a movement rather, relying upon the probable explanation of the mechanism by which it is produced advanced by Schiff, would not be possible in man, whose anterior extremities are not used in the act of progression. For it could only be by unilateral deviation of these, that the mammiferous quadruped, pushing forward the body by means of a regular movement of the posterior extremities, could accomplish the "circus-rider's movement," (*movimento di maneggio*,) or, as Moleschott describes it, "movement of the hands of a watch." But we should not hesitate upon this point if our case (like that of Stiebel, cited by Schiff himself,) did not present among other things this peculiarity, that the paralysis of the arm was more distinct and persistent than that of the leg. But it is easy to decide that this paralysis has no connection with that producing the lateral movements in animals, for it was complete in the left arm, while the right was entirely unaffected.

The rotatory movement, then, of our patient, is not comparable with the rotation of the mammifers upon their longitudinal axis, which, it is generally admitted, occurs in consequence of lesion of the middle cerebellar peduncles. These peduncles, according to Schiff, act in cruciform directions upon the rotating muscles of the vertebral column, and the direction of the movement of rotation is different according as the latter results from irritation or paralysis of the peduncular fibres. In the present case, the deviation being toward the left, we may suppose either paralysis of the muscles of the vertebral column of the right with prevalent action of those of the left side, or irritation of these last. Evidently we must accept the second hypothesis, because the movements made during the attack were of a convulsive character, and therefore the lesion should be sought for in the right cerebellar peduncle.

Other facts, however, are not consonant with such a diagnosis. The deviation of the eyes observed in our patient has not an exact correspondence with that observed by Schiff, following lesion of the middle cerebellar peduncle. Because, while the latter discovered that the eye of the injured side turned inward and downward, and that of the other outward and upward, the patient in our case presented a similar deviation in so far as that both eyes were turned to the left, while both were also turned upward; thus presenting a remarkable resemblance to the effects which Adamuk produced upon the ocular movements, by lesion of the corpora quadrigemina. The irritation of the anterior eminence of these bodies upon the right side, produces, according to this author, deviation of both eyes to the left, and vice versa. If the irritation is produced at a central point between the two anterior eminences toward their posterior limit, both eyes are still turned upward while the pupil is dilated. If now the excitation be continued for a somewhat longer time, the head is turned toward the side to which the eyes have been directed. These are precisely the phenomena presented by our patient. And moreover, a fact of great importance, we note the dilatation of the pupil, which represents a paralysis of the oculomotorius, associated with phenomena of irritation—a point to which Schiff does not refer in treating of ocular irritations by injury of the middle cerebellar peduncles. To this is to be added the circumstance that our patient after recovery, stated with great clearness that during his accesses he had never experienced any sensation of contortion of the spine, while yet he did perceive the dragging of the muscles upon the right side of the neck. It is clear that the movement of rotation of the eyes and head is most simply and clearly explained by the experiment of Adamuk.

Thus from the contraction with paralysis of the muscles of the face and limbs, we are led to establish a group of phenomena almost independent of those connected with the deviation of the eyes and head, because this second category of symptoms would be arrested at the period of transient irritation, making itself known by the attack, (excepting the paralysis of the *levator palpebrarum.*) And the graver lesion would be that associated with the muscles of the face and limbs. Possibly, also, the deviation of the eyes and head, if produced by irritation of the right anterior eminence of the corpora quadrigemina, would not have occurred except for lesions of the circulatory system, resulting from the action of a morbid principle at a point in the vicinity.

We should be therefore induced to look for the site of the lesion which produced the convulsive attack and the consequent hemiplegia, at a point more or less near the quadrigemellar tubercle of the right side.

The central paralysis of the facial leads to the admission of a morbid focus in the cerebral peduncle. In the present case, also, we have to deal with a simple paresis, precisely such as ordinarily occurs from lesion of the cerebral peduncle. The central paralysis of the facial would not, however, remove the possibility of a lesion of the pons varolii, situated above the point of decussation of the fibres of the same nerve, such as would also be sustained in lesion of the middle cerebellar peduncle, in consequence of the relation which exists between the two, according to the observations of Brown-Séquard and Magron. But the complexity and character of the symptoms presented in the case of our patient, as well as the absence of the phenomena which are not ordinarily wanting in lesion of the pons varolii and the cerebellar peduncles lead to the exclusion of these organs—at least we are thus brought to the exclusion of a lesion of the superficies of the cerebral lobes, involving the partial motor centres of Hitzig, Fritsch and Ferrier.

It remains, then, quite probable that the lesion is to be sought partly in the quadrigemellar body and partly in the cerebral peduncle. Yet the site of the lesion which produced the hemiplegic symptoms cannot be exactly established, since there is great variety in the method according to which primitive lesions may be diffused and give rise to secondary alterations. Especially is this true in the present case, where the nature of the lesion renders probable the existence of several primary points of injury, and gives room for numerous complications not only, on the one hand, in the cerebral substance, but also on the other, in the cerebral arteries, if not indeed in the meninges and cranial bones.

Still the interesting fact remains that this pathological history corresponds, in one essential part of its details, to a physiological fact recently established by the experimental method, and which thus renders possible a diagnosis of lesion of the quadrigemellar body. And though we have had the good fortune to cure our patient, and thus failed of the necroscopic corroboration which might have followed, perhaps our diagnostic reasoning may be profitable for others in establishing the relation between physiology and pathology.

Although the phenomena in question were at the outset of sudden occurrence, and accompanied by a total loss of consciousness, yet the peculiarity of the attacks, at first repeated only after a long repose, and subsequently after gradually decreasing periods, (with intervals of perfect health,) as well as the initiative symptoms which preceded the paralysis, enable us to exclude with readiness the occurrence of cerebral hæmorrhage. Capillary hæmorrhage can only be considered here as consecutive to embolism or thrombosis. Apoplectiform seizure, and the other disorders having a chronic course which apoplectiform disease may occasion, could result only from simple atheroma of the cerebral arteries. The condition of our patient rendered the last named hypothesis exceedingly improbable, nor would such a supposition be supported by similar alteration of the temporal artery and aorta, for here no such alteration existed.

We may then safely exclude thrombosis, embolism, and aneurism of the cerebral arteries. There remain merely the chronic lesions of the cerebral substance, in which a diagnostic distinction is attended with great difficulty, in consequence of the multiplicity of diseases capable of giving origin to apoplectiform attacks. These are, disseminated sclerosis, encephalitis, with chronic abscesses, ischæmia, and partial necrosis, and, lastly, tumors and syphilide of the brain.

In this hasty attempt to establish a differential diagnosis between all these diseases, some of which require but enumeration, we exclude all others in the concluding remark that from the first day of observation of this patient it seemed more than probable that we had to deal with a syphiloma. The symptoms, however, did not exactly correspond to either of the three forms of cerebral syphilide indicated by Heubner. Evidently they did not answer to the first, in which the epileptic attacks are accompanied by intellectual alterations. No less could they be assigned to the third form, in which the cerebral syphilide produces a progressive paralytic dementia.

The resemblance in this case was rather to the second form, which is characterized by true apoplectic attacks, with consecutive hemiplegia, generally involving the cerebral nerves, intercurrent states of somnolency and, very often, monolateral symptoms of irritation. Yet, the first two attacks only could be described as really apoplectic, in which, it is true, loss of consciousness was not wanting, and the hemiplegia required several days in order to disappear. The last attacks had rather the aspect of epileptiform convulsions without loss of consciousness, which may occur in the more irregular forms of epilepsy. But the phenomena of monolateral irritation, and the lesion of the cerebral nerves, are sufficiently marked to enable us to inscribe our case in the second category.

The syphilitic nature of the lesion being admitted, it is easy to explain all the subsequent phenomena, including the diffusion of the excitation even to some of the muscles of the right side by the possibility of arterial complication. The latter, possibly, produced the first attack by the occurrence of hæmorrhage, while the latter seizures could have resulted from a circumscribed encephalitis as the sole morbid condition without the need of the syphiloma. And this appears quite probable from the lack of the initial symptoms of tumor, and especially from the absence of cephalalgia.

The diagnosis of cerebral syphilide being thus established the treatment was naturally determined; and the criterion afforded by the therapy confirmed the diagnosis in a most luminous manner. When Ricolfi first presented himself at the clinic, he had already taken for a considerable time the bromide of potassium in average doses of 8 grammes daily. After his admission it was decided to make further trial of this salt in larger doses. At the same time chloral was exhibited, and hypodermic injections of the sulphate of atropia were employed, in the effort to procure temporary respite from the tumult of motor disturbances. Recognizing, however, the complete inutility of this treatment, a few days after the diagnosis was established as at least probable, recourse was had to mercury, which was at first introduced into the system in the form of calomel by hypodermic injections. Afterward, the biniodide was employed in pillular form. Soon the iodide of potassium was added to the mercurial, and, under this anti-syphilitic treatment, within a short space of time, and in a marvellous manner, the attacks began to diminish in frequency and intensity, and finally completely ceased. The patient, in fact, left the clinic on the 15th of April in a condition of perfect health.

